# Stability and Formulation of Erlotinib in Skin Creams

**DOI:** 10.3390/molecules27031070

**Published:** 2022-02-05

**Authors:** David Nguyen, Philippe-Henri Secrétan, Camille Cotteret, Emmanuelle Jacques-Gustave, Céline Greco, Christine Bodemer, Joel Schlatter, Salvatore Cisternino

**Affiliations:** 1 Service Pharmacie, APHP, Hôpital Necker-Enfants Malades, F-75015 Paris, France; david.nguyen@aphp.fr (D.N.); philippe-henri.secretan@u-psud.fr (P.-H.S.); camille.cotteret@aphp.fr (C.C.); emmanuelle.jacques-gustave@aphp.fr (E.J.-G.); 2 Matériaux et santé, Université Paris-Saclay, F-92296 Châtenay-Malabry, France; 3 Department of Pain and Palliative Care Unit, APHP, Hôpital Necker-Enfants Malades, F-75015 Paris, France; celine.greco@aphp.fr; 4 IMAGINE Institute, INSERM, U1163, Université de Paris, F-75015 Paris, France; christine.bodemer@aphp.fr; 5 Reference Center for Genodermatoses (MAGEC), Department of Dermatology, APHP, Hôpital Necker-Enfants Malades, F-75015 Paris, France; 6 Pharmacie, APHP, Hôpital Paul-Doumer, F-60140 Liancourt, France; 7 INSERM UMR_S1144, Optimisation Thérapeutique en Neuropsychopharmacologie, Université de Paris, F-75006 Paris, France

**Keywords:** dermatology, drug repurposing, erlotinib, rare disease, stability indicating, Olmsted, skin cancer, topical

## Abstract

Recent studies have highlighted the benefit of repurposing oral erlotinib (ERL) treatment in some rare skin diseases such as Olmsted syndrome. The use of a topical ERL skin treatment instead of the currently available ERL tablets may be appealing to treat skin disorders while reducing adverse systemic effects and exposure. A method to prepare 0.2% ERL cream, without resorting to a pure active pharmaceutical ingredient, was developed and the formulation was optimized to improve ERL stability over time. Erlotinib extraction from tablets was incomplete with Transcutol, whereas dimethyl sulfoxide (DMSO) allowed 100% erlotinib recovery. During preliminary studies, ERL was shown to be sensitive to oxidation and acidic pH in solution and when added to selected creams (i.e., Excipial, Nourivan Antiox, Pentravan, and Versatile). The results also showed that use of DMSO (5% *v*/*w*), neutral pH, as well as a topical agent containing antioxidant substances (Nourivan Antiox) were key factors to maintain the initial erlotinib concentration. The proposed ERL cream formulation at neutral pH contains a homogeneous amount of ERL and is stable for at least 42 days at room temperature in Nourivan cream with antioxidant properties.

## 1. Introduction

Erlotinib (ERL) is a very lipophilic drug (clog P ~ 3.2) and potent epidermal growth factor receptor (EGFR) inhibitor (IC_50_ ~ 2 nM) currently marketed as tablets (Tarceva^®^) for the treatment of EGFR mutation-positive non-small-cell lung cancer [1,2]. Based on its mechanism of action, the repurposing of this tyrosine kinase inhibitor drug has been studied in the treatment of local skin disorders such as palmoplantar keratoderma in selected patients with Olmsted syndrome [3], cutaneous squamous cell carcinoma [4], and psoriasis [5]. Indeed, when treated with ERL administered orally, the authors reported an improvement of palmoplantar keratoderma in patients with Olmsted syndrome [3].

Localized diseases are preferably managed using local treatment procedures. Topical forms are often preferred as in many cases they allow an improved exposition and contact with the drug and a lower systemic exposure, resulting in a decrease of the adverse effect and a better benefit/risk ratio [6,7,8,9]. The use of topical ERL instead of or in addition to the oral ERL tablets may be especially interesting in the control of local skin diseases, as the oral drug is known to induce side effects such as folliculitis, diarrhea, paronychia, fatigue, and hair changes [10]. However, since the change from a solid form to a solubilized form induces a significant change in the chemical environment, the drug is often much more prone to chemical instability [11]. As ERL is known to degrade upon hydrolytic and oxidative stress, with degradation products having been detected in the presence of hydrochloric acid (HCl), sodium hydroxide (NaOH), and hydrogen peroxide (H_2_O_2_) [12,13], the impact of formulation change needs to be assessed.

As a result, this study was conducted to design a stable ERL skin cream. To make this formulation more feasible without access to ERL marketed pharmaceutical pure powder, the available form of pharmaceutical grade erlotinib (e.g., tablets), commonly used excipients, and easily reproducible preparation conditions were chosen. An extraction protocol to obtain pharmaceutical grade ERL was developed. Preliminary studies were performed to highlight conditions where the stability of ERL is favored. The stability of the optimized formulation was assessed by following the content of erlotinib by using a validated stability-indicating chromatographic method. 

## 2. Results

### 2.1. Preliminary Tests

#### 2.1.1. Intrinsic Stability of ERL

The stress testing protocol, following the recommendations of ICH Q1A, and the associated results are developed in the Supplementary Materials. ERL decrease was very fast under oxidative conditions (3% H_2_O_2_) and simulated light conditions (ICH Q1B light conditions), where about 9% and 30% loss were observed after 8 h and 24 h of exposure, respectively (Table 1). Under acidic (0.1 M HCl) and alkaline (0.1 M NaOH) conditions, the remaining amount of ERL after 21 days was 97.7% and 98.8%, respectively. As a counterpart, 3, 2, 8, and 6 degradation products were detected, respectively, under acidic, alkaline, oxidative, and photolytic conditions (Table 1).

#### 2.1.2. Method Validation

The method was validated in terms of specificity, linearity, and accuracy as per the ICH Q2 guidelines [14]. Chromatograms (Figures S1–S4) showed the good separation of ERL from its degradation products. Regarding assay results, linearity was observed over the concentration range from 80 to 120 µg·mL^−1^ (y = 1.50·x–4.91; r^2^ = 0.995). The confidence interval of the accuracy was [98.4%; 101.4%] and included the value 100%. Precision (coefficients of variation) was below 5%. The limits of detection and quantification were 3.6 and 10.8 µg·mL^−1^, respectively.

#### 2.1.3. Extraction of ERL from the Tablets

**Figure 1:**
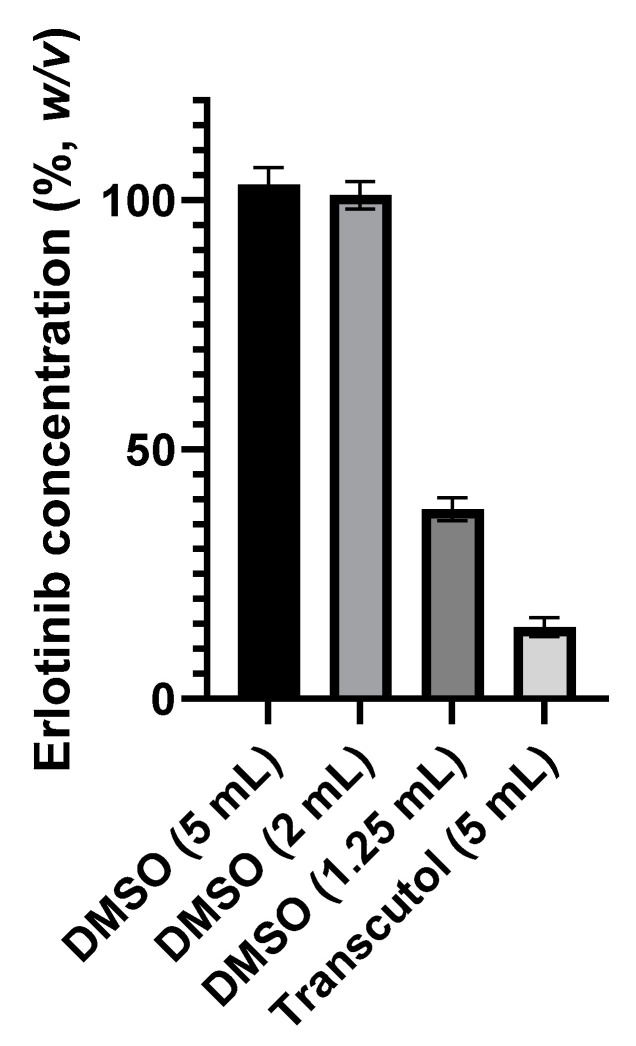
Erlotinib 100 mg tablet extraction recovery (%) yield as a function of DMSO (5; 2; 1.25 mL) and Transcutol^®^ (5 mL) volume. One extract was obtained for each condition and was analyzed three times.

Tablet solubilization tests were carried out in Transcutol^®^ and dimethyl sulfoxide (DMSO). The use of DMSO gave a yield of 103.1 ± 3.4%, while the use of Transcutol^®^ only gave a yield of 14.4 ± 4.6% (Figure 1). The best of the two chosen compounds for ERL solubilization is therefore DMSO (*p* < 0.0001).

Tests were then carried out with different volumes of DMSO to solubilize the tablets and to assess what would be the minimum volume to be used to obtain an at least 95% yield.

Using 2 mL and 5 mL of DMSO to solubilize a 100 mg tablet resulted in, theoretically, 50 mg·mL^−1^ and 20 mg·mL^−1^ extracts, respectively, in which ERL assays were above 95% (Figure 1). Thus, to limit drug wastage, both these extracts were retained for the preliminary stability tests of the cream.

#### 2.1.4. Preliminary Stability Tests

The preliminary stability consisted of blending four ready-to-use formulations with extracts of DMSO of two different concentrations of ERL (20 mg·mL^−1^ and 50 mg·mL^−1^) to obtain the final concentration of ERL of 0.1% (*w*/*w*). Thus, the formulations used in the preliminary tests, respectively, contained either 5% (*v*/*w*) or 2% (*v*/*w*) DMSO.

**Figure 2:**
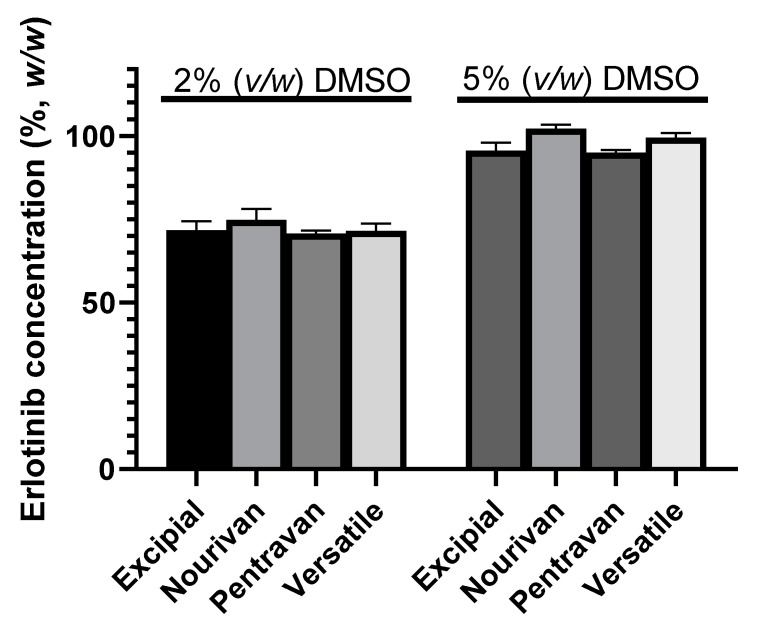
Erlotinib concentration (%, *w*/*w*) after one month of storage at ambient temperature and as a function of the volume of the extract of DMSO and of the four ready-to-use cream vehicles: Excipial^®^, Nourivan Antiox^®^, Pentravan^®^, Versatile^®^. Three extracts were obtained for each condition, and each extract was analyzed once.

The recoveries of ERL in the creams after 1 month of storage at ambient temperature are gathered in Figure 2.

Two-way ANOVA showed that both the amount of DMSO (*p* < 0.0001) and the choice of ready-to-use formulation (*p* = 0.001) had effects on the assay value, but interaction was found to be not significant (*p* = 0.218). The highest assay value (mean = 102.3%; SD = 1.1%) was obtained when the ready-to-use formulation was Nourivan Antiox^®^ and when the formulation contained 5% DMSO. When gathering all the results of the erlotinib concentration (%, *w*/*w*) of the creams prepared as a function of DMSO content, the *t*-test showed a significant difference (*p* < 0.0001) between the group containing 2% DMSO (72.2 ± 2.6%, *n* = 12) to that containing 5% DMSO (98.1 ± 3.4%, *n* = 12).

### 2.2. Stability of ERL under Optimized Conditions of Preparation

The knowledge acquired through the preliminary experiments, discussed in Section 3, enabled us to propose optimized conditions of preparation for a topical ERL at 0.2% (*w*/*w*). This consisted of extracting ERL tablets in DMSO to obtain a 20 mg·mL^−1^ extract followed by incorporating the extract in appropriate amount of Nourivan Antiox^®^. Six batches were prepared: three without altering the pH of the batches (pH ~ 3.5), and three batches with the pH of the blend adjusted to ~7.0 with sodium hydroxide.

**Figure 3:**
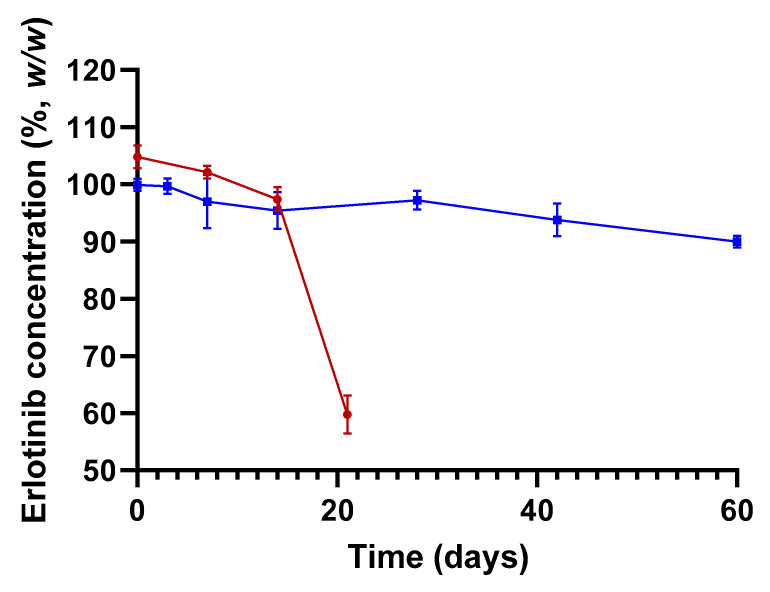
Stability of ERL 0.2% formulated in Nourivan Antiox^®^ at pH ~ 3.5 (red curve) and pH ~ 7.0 (blue curve). The lower (90%, *w*/*w*) and upper (110%, *w*/*w*) specifications are represented by the dotted grey horizontal lines. Each data point and associated standard deviation were obtained by analyzing the corresponding three batches of cream separately.

Without pH change of the blend (pH ~ 3.5), after 21 days, the mean assay of ERL was 57.0 ± 3.2% (Figure 3).

When adjusting the pH of the blend to a neutral value, the results complied with the specification range of 90.0–110.0% from release to 42 days (Figure 3). Thus, in terms of assay, for at least one month, this preparation corresponded to the limit acceptable for most compounded preparations that has been established by the US Pharmacopeia (USP) [15].

**Figure 4:**
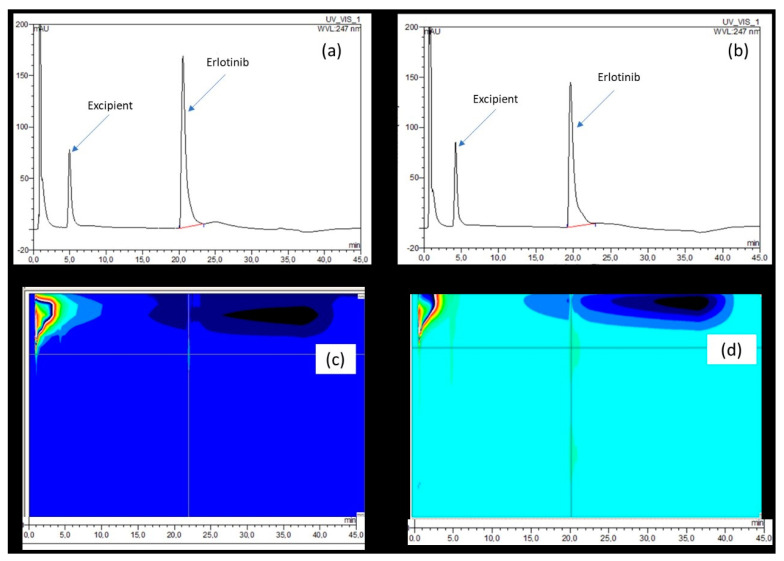
Typical chromatograms recorded at 247 nm on the day (**a**) and 42 days after preparation (**b**) and the corresponding 3D chromatograms (**c**,**d**) recorded from 200 to 800 nm.

At both the wavelength used for the detection of ERL (247 nm) and when using DAD (200–800 nm), no difference could be detected between the signal obtained at the beginning (Figure 4, inset a and c) and after 42 days of storage (Figure 4, inset b and d).

## 3. Discussion

In the absence of available topical ERL cream as well as ERL pharmaceutical grade pure powder, one possible way to obtain a 0.2% ERL cream (*w*/*w*) is by crushing the corresponding number of tablets and blending the resulting powder with a ready-to-use vehicle. This approach is not suitable, as the powder of the tablet gives a grainy appearance to the cream, associated with a partial ERL dissolution, and an unacceptable cream texture. It was first necessary to find reproducible conditions to solubilize and extract ERL from the ERL marketed tablets. The extraction of ERL, a very lipophilic drug, from tablets was carried out in two solvents (i.e., DMSO, Transcutol^®^) suitable for topical formulation and with different volumes so as to optimize the amount of solvent used.

After having found the appropriate conditions to obtain sufficient and reproducible extraction yields with DMSO, the choice of an appropriate vehicle to administer the drug topically was the second aim of this work. This choice relied on considerations related to the patient’s tolerance and the drug’s stability. Based on the advice of a clinician, four ready-to-use formulations were chosen for the preliminary tests. Knowledge of the intrinsic stability of ERL and the results of the preliminary stability tests were used to determine conditions to obtain an ERL skin topical formulation that is as stable as possible.

During the preliminary studies, the only formulations which kept sufficient amounts of ERL were those prepared by the use of DMSO, i.e., the formulation including the highest quantity of DMSO. This result may be explained by the fact that ERL is sensitive to oxidative degradation as highlighted by the stress testing study (Table 1 and Figure S3), and that DMSO reduces the risk of oxidation [16]. Further, for a given amount of DMSO (i.e., 2% or 5% *v*/*w*) the highest assay results for ERL were always obtained with Nourivan Antiox^®^, confirming that using an environment protecting ERL from oxidation may be necessary to keep the ERL amount at the appropriate level.

However, when following the stability of 0.2% ERL (*w*/*w*) formulated in Nourivan Antiox^®^, the physico-chemical environment of ERL again seemed to require improvement in so far that only 57% of the ERL remained in the cream after three weeks. Based on the results of the pH of the formulation (pH ~ 3.5), and on the propensity of ERL to degrade upon acidic stress [12], the loss of ERL in this formulation may be due to the low/acidic pH. Thus, the optimization of the stability consisted of increasing the pH of the formulation to 7.0, a pH close to the skin and better tolerated by the tissues. Using these optimized conditions of preparation on three batches, the results complied with the specification range of 90.0–110.0% from release to 42 days. Thus, the preliminary results of this study may pave the way to ERL creams with improved stability and responding to the requirements of a marketing authorization (see ICH Q1 A&B).

Another aspect to consider is the formation of degradation products, as it is recommended to assess the toxicity and risk related to their presence in pharmaceutical products [17]. In that sense, we investigated if degradation products were detected in the optimized preparation. After 42 days of storage at room temperature, no degradation products were quantitatively formed as the purity profile did not differ to that just after preparation (Figure 4). When using the proposed conditions, the risk of a toxic effect resulting from ERL degradation products seems limited. One critical aspect of the cream is the amount (5% *v*/*w*) of DMSO. According to the ICH Q3C classification of solvents [18], DMSO belongs to the least toxic class of solvent (i.e., class 3), which available data indicate are less toxic in acute or short-term studies and negative in genotoxicity studies. DMSO has a low toxic potential and it is considered in ICH Q3C that amounts of DMSO of 50 mg per day or less are acceptable without justification. Based on the drug formula, one could use 1 g of the topical formulation per day without justification.

## 4. Materials and Methods

### 4.1. Preparation of Test Creams

ERL creams were prepared by extracting Tarceva^®^ tablets containing 100 mg of ERL hydrochloride (Roche, Zurich, Switzerland) with pharmaceutical grade dimethyl sulfoxide (DMSO, WAK-Chemie, Steinbach, Germany). Amounts of 2.0 or 5.0 mL of DMSO were added to a 100 mg ERL crushed tablet. These suspensions were sonicated for 60 min and centrifugated at 10,000× *g* rpm for 5 min. After assay of the supernatant, this extract was added to four different topical vehicles: Excipial^®^ hydrocream (Gallderma, Toulouse, France), Nourivan Antiox^®^, Pentravan^®^, and Versatile^®^ (Fagron, Thiais, France) and manually stirred to obtain the target concentration of ERL. The mixtures were transferred into empty aluminum ointment tubes with internal varnish (COOPER, Melun, France), sealed, and stored at room temperature (20 ± 2 °C). The creams were prepared under laminar flow to minimize the risk of microbial contamination and reduce caregivers’ exposure to ERL. The pH of the cream was checked with digital SevenEasy S20 pH meter (Mettler-Toledo, Viroflay, France) equipped with a calibrated InLab^®^ Expert Pro-ISM pH sensor (Mettler-Toledo).

### 4.2. Chromatographic Conditions

Analysis was performed by a high-performance liquid chromatograph (HPLC) (U3000 Ultimate, Thermo Fisher Scientific, Courtaboeuf, France) coupled to diode array detection (DAD). The conditions proposed by Mahajan et al. and Pujeri et al. were taken as a starting point for optimization [12,19]. A Zorbax^®^ C18 column (dimensions, 75 by 4.6 mm; particle size, 3.5 µm) was used for separation. The mobile phase consisted of methanol (A) and formate buffer solution (B: 10 mM, pH adjusted to 3.5) in gradient mode (Tmin/A:B; T0/35:65; T9/35:65; T35/65:35; T40/35:65). The column temperature, the flow rate, and the detection for quantification were set at 25 ± 2 °C, 1.0 mL·min^−1^, and 246 nm, respectively. Data acquisition (e.g., peak time, area) was carried out using Chromeleon^®^ software (v6.80, Thermo Fisher Scientific).

### 4.3. Assay Method Validation

Forced degradation studies were carried out to demonstrate the stability-indicating capability of the HPLC method. Four stress conditions were applied on ERL: acidic and alkaline hydrolysis and oxidative and photolytic conditions. To that end, a stock solution of ERL in DMSO (200 µg·mL^−1^) was diluted in equal parts with aqueous 0.2 M HCl, 0.2 M NaOH, or 6% H_2_O_2_, and ultrapure water, respectively. For photolytic stress studies, the working solution was exposed to artificial weathering light, using a xenon test chamber Q-SUN Xe-1 (Q-Lab Corporation, Saarbrücken, Germany) operating in window mode and complying with ICH Q1B recommendations. The light beam, presenting a characteristic spectrum ranging from 300 to 800 nm, was set at an intensity of 1.50 W/m^2^ at 420 nm. Forced degradation conditions are shown in Table 2.

Regarding assay method validation, calibration standard and quality control (QC) stock solutions of ERL hydrochloride (analytical grade, Sigma Aldrich, St Quentin Fallavier, France) were prepared in DMSO independently. A working solution of ERL were prepared daily by the dilution of stock solutions with the mobile phase to prepare calibration standard (80, 90, 100, 110, and 120 µg·mL^−1^) and QC samples (100 µg·mL^−1^).

### 4.4. Stability Procedures

To carry out an assay of the cream, the sample of cream from each tube was weighed in a volumetric flask and DMSO was added to obtain a theoretical concentration of about 100 µg·mL^−1^. The content was then stirred magnetically for 30 min and sonicated for another 30 min to enable a complete solubilization of the ERL of the formulation. After centrifugation (10,000× *g* rpm, 5 min), the supernatant was collected and assayed.

The erlotinib concentration (%, *w*/*w*) of ERL in the topical formulation was obtained by the following formula:

(1) $$\frac{r\left(f\right)}{r\left(s\right)}\cdot 100$$

where *r*(*f*) and *r*(*s*) are the chromatographic peak responses obtained for the formulation assay solution and for the standard solution (100 µg·mL^−1^), respectively.

Regarding the stability protocol of the cream, a preliminary physicochemical stability study was carried out with 0.1% ERL cream stored at room temperature (20 ± 2 °C) to determine the best topical vehicle and DMSO volume used for extraction (2% *v*/*w* or 5% *v*/*w* in the final formulation). Three cream tubes were assayed on days 0, 28, and 84.

A second study with the optimum formulation parameters of topical vehicle and DMSO volume was executed. Three batches of 0.2% ERL cream were adjusted or not to a neutral pH with sodium hydroxide and stored at room temperature. They were assayed on days 0, 14, 28, 42, and 56.

## 5. Conclusions

The proposed method of preparation enables a homogeneous formulation to be obtained by the use of ERL tablets. This formulation contains more than 90% of the drug after 42 days at room temperature. The provided preparation protocol may be followed if the use of topical ERL to treat cutaneous ailments is investigated. The use of this ERL cream formulation may be proposed in clinical trial to evaluate its action on local skin disorders while limiting the unwanted systemic effects.

## Figures and Tables

**Table 1:** Typical results of the forced degradation study.

Condition	% Recovery of Erlotinib	Duration of Exposure	Number of Degradation Products Detected
Acidic (0.1 M HCl)	97.7	21 days	3
Alkaline (0.1 M NaOH)	98.8	21 days	2
Oxidative stress (3% H_2_O_2_)	91.0	8 h	8
Photolytic (ICH Q1B light conditions)	60.5	24 h	6

**Table 2:** Forced degradation conditions.

Conditions	Analysis Time	Temperature
Acidic (0.1 M HCl)	Day 0, 1, 7, 14, 21	50 °C
Alkaline (0.1 M NaOH)	Day 0, 1, 7, 14, 21	50 °C
Oxidative stress (3% H_2_O_2_)	0, 8, 24, 48 h	20 °C
Photolytic (ICH Q1B lamp)	0, 4, 8 h	20 °C

## Data Availability

Data from the study are available from the corresponding author upon reasonable request.

## Supplementary Materials

The Supplementary Materials are available online. Figure S1: HPLC chromatograms of ERL in acidic conditions, Figure S2: HPLC chromatograms of ERL in alkaline conditions, Figure S3: HPLC chromatograms of ERL in oxidative conditions, Figure S4: HPLC chromatograms of ERL in photolytic conditions.

## References

[1] Tsao M., Sakurada A., Cutz J.-C., Zhu C., Kamel-Reid S., Squire J., Lorimer I., Zhang T., Liu N., Daneshmand M. (2005). Erlotinib in Lung Cancer—Molecular and Clinical Predictors of Outcome. N. Engl. J. Med..

[2] Rosell R., Carcereny E., Gervais R., Vergnenegre A., Massuti B., Felip E., Palmero R., Garcia-Gomez R., Pallares C., Sanchez J.M. (2012). Erlotinib versus standard chemotherapy as first-line treatment for European patients with advanced EGFR mutation-positive non-small-cell lung cancer (EURTAC): A multicentre, open-label, randomised phase 3 trial. Lancet Oncol..

[3] Greco C., Leclerc-Mercier S., Chaumon S., Doz F., Hadj-Rabia S., Molina T., Boucheix C., Bodemer C. (2020). Use of Epidermal Growth Factor Receptor Inhibitor Erlotinib to Treat Palmoplantar Keratoderma in Patients with Olmsted Syndrome Caused by TRPV3 Mutations. JAMA Dermatol..

[4] Gold K.A., Kies M.S., William W.N., Johnson F.M., Lee J.J., Glisson B.S. (2018). Erlotinib in the treatment of recurrent or metastatic cutaneous squamous cell carcinoma: A single-arm phase 2 clinical trial. Cancer.

[5] Overbeck T.R., Griesinger F. (2012). Two Cases of Psoriasis Responding to Erlotinib: Time to Revisiting Inhibition of Epidermal Growth Factor Receptor in Psoriasis Therapy?. J. Dermatol..

[6] Kopsky D., Bhaskar A., Zonneveldt H., Keppel Hesselink J. (2019). Topical loperamide for the treatment of localized neuropathic pain: A case report and literature review. J. Pain Res..

[7] Assmann T., Homey B., Ruzicka T. (2001). Topical tacrolimus for the treatment of inflammatory skin diseases. Expert Opin. Pharmacother..

[8] Choi F.D., Juhasz M.L., Mesinkovska N.A. (2019). Topical ketoconazole: A systematic review of current dermatological applications and future developments. J. Dermatol. Treat..

[9] Bouchand C., Nguyen D., Secretan P.-H., Vidal F., Guery R., Auvity S., Cohen J.F., Lanternier F., Lortholary O., Cisternino S. (2020). Voriconazole topical cream formulation: Evidence for stability and antifungal activity. Int. J. Antimicrob. Agents.

[10] Becker A., van Wijk A., Smit E.F., Postmus P.E. (2010). Side-Effects of Long-Term Administration of Erlotinib in Patients with Non-small Cell Lung Cancer. J. Thorac. Oncol..

[11] Lieberman H., Vemuri N.M. (2015). Chemical and Physicochemical Approaches to Solve Formulation Problems. The Practice of Medicinal Chemistry.

[12] Mahajan A.A., Miniyar P.B., Patil A.S., Waghmare R.U., Patil J.J., Mohanraj K., Tiwari R.N. (2014). Separation, Identification, and Characterization of Degradation Products of Erlotinib Hydrochloride Under ICH-Recommended Stress Conditions by LC, LC-MS/TOF. J. Liq. Chromatogr. Relat. Technol..

[13] Negreira N., Regueiro J., de Alda M.L., Barceló D. (2015). Degradation of the anticancer drug erlotinib during water chlorination: Non-targeted approach for the identification of transformation products. Water Res..

[14] ICH Expert Working Group (1994). ICH Q2 (R1) Validation of Analytical Procedures: Text and Methodology.

[15] Allen L.V., Bassani G.S., Elder E.J., Parr A.F. (2014). Strength and Stability Testing for Compounded Preparations. US Pharmacop..

[16] Sanmartín-Suárez C., Soto-Otero R., Sánchez-Sellero I., Méndez-Álvarez E. (2011). Antioxidant properties of dimethyl sulfoxide and its viability as a solvent in the evaluation of neuroprotective antioxidants. J. Pharmacol. Toxicol. Methods.

[17] Melo S.R.D.O., Homem-De-Mello M., Silveira D., Simeoni L.A. (2014). Advice on Degradation Products in Pharmaceuticals: A Toxicological Evaluation. PDA J. Pharm. Sci. Technol..

[18] ICH Expert Working Group (2021). ICH Guidelines Q3C (R8) on Impurities, Guidelines for Residual Solvents.

[19] Pujeri S.S., Khader A.M.A., Seetharamappa J. (2009). Validated Stability-Indicating Chromatographic Method for the Assay of Erlotinib Active Pharmaceutical Ingredient. Anal. Lett..

