# Supplementary material for: Stability and Formulation of Erlotinib in Skin Creams
Source: Molecules. 2022 Feb 5;27(3):1070. doi: 10.3390/molecules27031070 (PMC8839675; doi:10.3390/molecules27031070)
Supplement: Supplementary file 1 [file molecules-27-01070-s001.zip › molecules-1579234-supplementary.pdf]

Stability and Formulation of Erlotinib in Skin Creams

David Nguyen [1,†], Philippe-Henri Secrétan [1,2,†], Camille Cotteret [1], Emmanuelle Jacques-Gustave [1], Céline Greco [3,4], Christine Bodemer [4,5], Joel Schlatter [1,6,*,‡] and Salvatore Cisternino [1,7,*,‡]

Supplemental Results of the stress testing study

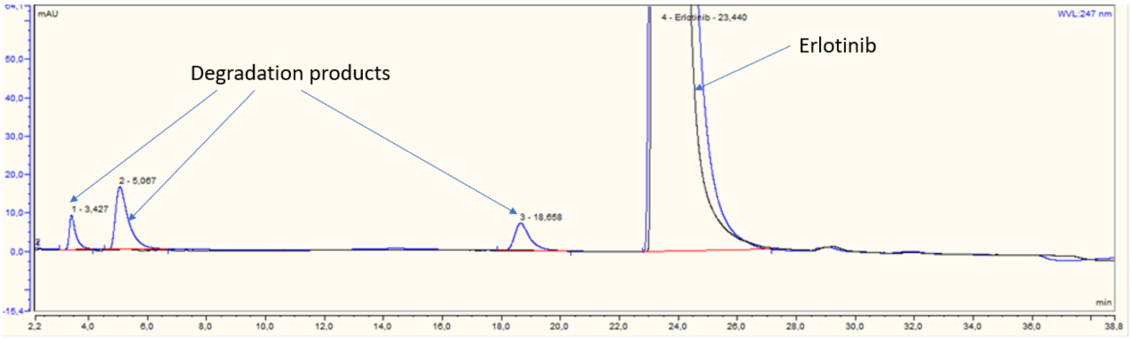

Figure S1: HPLC chromatograms of ERL in acidic conditions. Black chromatogram: the solution analyzed just after exposure to acidic conditions. Blue chromatogram: same solution analyzed after 21 days of exposure to acidic conditions.

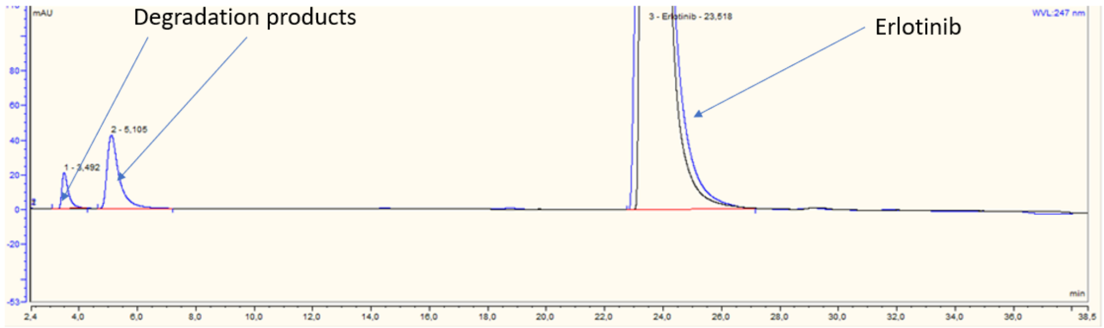

Figure S2: HPLC chromatograms of ERL in alkaline conditions. Black chromatogram: the solution analyzed just after exposure to alkaline conditions. Blue chromatogram: same solution analyzed after 21 days of exposure to alkaline conditions.

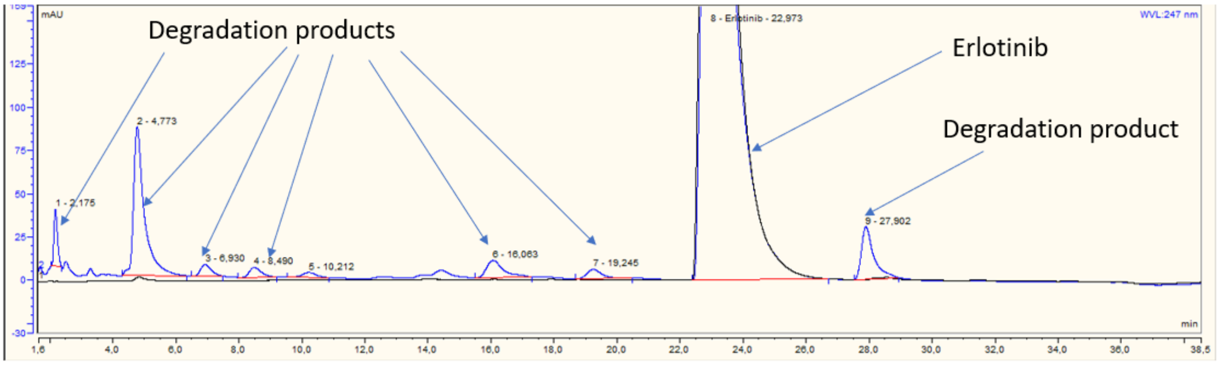

Figure S3: HPLC chromatograms of ERL in oxidative conditions. Black chromatogram: the solution analyzed just after exposure to oxidative conditions. Blue chromatogram: same solution analyzed after 8 hours of exposure to oxidative conditions.

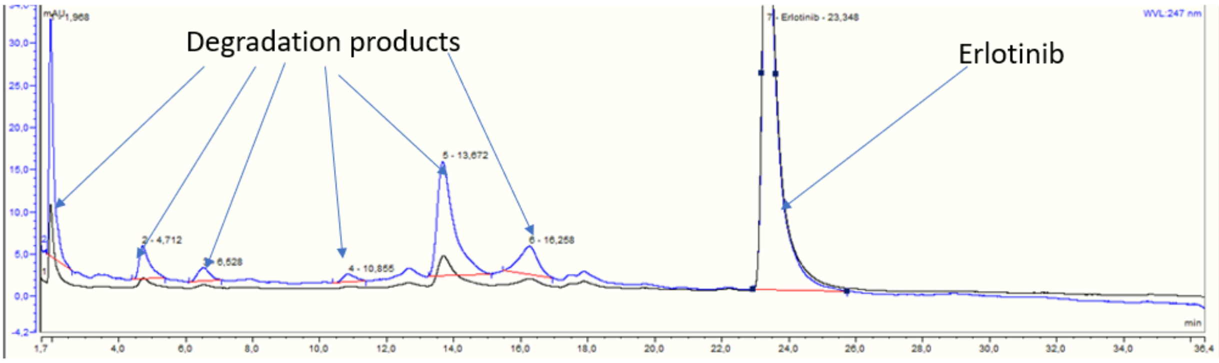

Figure S4: HPLC chromatograms of ERL in photolytic conditions. Black chromatogram: the solution analyzed prior to exposure to photolytic conditions. Blue chromatogram: same solution analyzed after 24 hours to simulated light.
